# Chronic Prostatic Infection and Inflammation by *Propionibacterium acnes* in a Rat Prostate Infection Model

**DOI:** 10.1371/journal.pone.0051434

**Published:** 2012-12-11

**Authors:** Jan Olsson, Johanna Bergh Drott, Lovisa Laurantzon, Oscar Laurantzon, Anders Bergh, Fredrik Elgh

**Affiliations:** 1 Department of Clinical Microbiology, Virology, Umeå University, Umeå, Sweden; 2 Department of Medical Biosciences, Pathology, Umeå University, Umeå, Sweden; University of Illinois at Chicago College of Medicine, United States of America

## Abstract

Chronic inflammation in the prostate, seen as infiltration of inflammatory cells into the prostate gland in histological samples, affects approximately half the male population without indication of prostate disease, and is almost ubiquitous in patients diagnosed with benign prostate hyperplasia and cancer. Several studies have demonstrated the Gram-positive bacterium *Propionibacterium acnes* to be frequently present in prostate tissue from men suffering from prostate disease. *P. acnes* has been shown to be associated with histological inflammation in human prostatectomy specimens, and also to induce strong inflammatory response in prostate-derived tissue culture models. The present paper describes a rat model for assessment of the pathogenic potential of *P. acnes* in prostate. Prostate glands of Sprague Dawley rats (n = 98) were exposed via an abdominal incision and live *P. acnes* or, in control rats, saline were injected into the ventral and dorso-lateral lobes. Rats were sacrificed 5 days, 3 weeks, 3 months and 6 months post infection, and prostate tissue was analyzed for bacterial content and histological inflammation. Rat sera were assessed for levels of CRP and anti-*P. acnes* IgG. Live *P. acnes* could be recovered from the dorso-lateral lobes up to 3 months post infection, while the ventral lobes were cleared from bacteria at that time. In samples up to 3 months post infection, the dorso-lateral lobes exhibited intense focal inflammation. CRP and IgG levels were elevated throughout the span of the experiment, and reached maximum levels 3 weeks and 3 months post infection, respectively. We show that *P. acnes* have the potential to cause chronic infection in previously healthy prostate, and that the infection has potential to cause chronic histological inflammation in the infected tissue. The high prevalence of *P. acnes* in human prostate tissue calls for resolution of pathogenic details. The present rat model suggests that complications such as chronic inflammation may be induced by *P. acnes* infection.

## Introduction

Chronic inflammation in the prostate, seen as infiltration of inflammatory cells into the prostate gland in histological samples, affects approximately half the male population without indication of prostate disease [1], and is almost ubiquitous in patients diagnosed with benign prostate hyperplasia and cancer [2], [3]. Accumulating evidence suggests that prostatic inflammation contributes significantly to the etiology of prostate cancer [4], [5] as well as benign prostatic hyperplasia (BPH) [1], [6]. Bacterial colonization and infection of the prostate have been implicated as contributing to the initiation and maintenance of chronic inflammation [7], [8], [9]. Asymptomatic or subclinical bacterial infections in the prostate appear to be relatively common, yet largely under-diagnosed [10], [11]. Several studies have demonstrated high prevalence rates of the Gram-positive bacterium *Propionibacterium acnes (P. acnes )*in prostate tissue from men diagnosed with prostate disease [12], [13], [14]. Serum titres of *P. acnes* antibodies correlate positively with PSA in cancer-negative patients [15], thus indicating *P. acnes* involvement in prostatic inflammation. Furthermore, *P. acnes* has been shown to be associated with histological inflammation in human prostatectomy specimens [13] and to induce a strong inflammatory response in prostate derived tissue culture models [14], [16]. However, well-characterized models of acute and chronic prostate infection are yet to be developed. The present paper describes a rat model of prostatic *P. acnes* infection for the assessment of acute and chronic infection/inflammation in wild-type animals.

## Materials and Methods

### *Propionibacterium acnes* Cultivation

Two batches of *Propionibacterium acnes* bacteria were cultivated from frozen stock in BHI +5% horse serum at 37°C under microaerophilic conditions; type 1A (CCUG 41530) and a mixture of four human prostate isolates, two of type 1 and two of type 2 [17], respectively. Exponentially growing bacteria were collected after two passages in fresh medium, washed with sterile saline by centrifugation and resuspended into saline at a density of 1·10^7^ CFU/µl.

### Animals and Animal Treatment

Adult male Sprague Dawley rats (age 3–4 months, weight: 400–500 g) (n = 98) (B&K, Stockholm Sweden) were anesthetized with pentobarbital (50 mg/kg) and an incision was made in the lower abdomen to expose the prostate. *Propionibacterium acnes* (5 µl) (5·10^7^ CFU) (type 1A in animals to be infected for 5 days and 3 weeks, respectively, and prostate isolate-*P. acnes* mixture in animals to be infected for 3 weeks, 3 months and 6 months, or saline (5 µl) was injected with a Hamilton syringe into the left ventral prostate (VP_L_) and into the dorso-lateral prostate (DLP) lobes. After 5 days, 3 weeks, 3 months and 6 months, blood samples were collected by cardiac puncture and, subsequently, animals were sacrificed. Left ventral (VP_L_), right ventral (VP_R_), and dorso-lateral (DLP) prostate lobes were excised and treated for bacterial counts or fixed in formalin for subsequent histological analysis.

### Ethics

The rats were maintained at the animal facility at Umeå University and all experiments involving animals were approved by the local Animal Review Board (Umeå, Sweden) (approval Ids: 2008/293, date:081029, A81-06, date:060818, A82-06, date:060818). All surgery was performed under sodium pentobarbital anesthesia, and all efforts were made to minimize suffering.

### Hematoxylin/Eosin- and Immunofluorescence Stainings

Sample tissue was fixed in formalin, dehydrated and embedded in paraffin. Four micron thick sections were deparaffinized and rehydrated. The tissue sections were stained with hematoxylin & eosin according to standard procedures. Tissue was examined for histological inflammation (see below) with an Olympus AX-70 microscope and documented with an ALTRA 20 CCD camera. For IF, deparaffinized sections were antigen-retrieved by boiling in citrate buffer (10 mM, pH 6.0) at 2 atm for 1 h. Following blocking with 1% BSA in PBS, slides were incubated with *P. acnes* antiserum diluted 1∶1000 in blocking solution for 1 h. Slides were washed in PBS and incubated for 1 h with goat anti-rabbit monoclonal antibodies labeled with alexa 488 (Invitrogen) diluted 1∶1000 in blocking buffer. Following washing and dehydration, the slides were mounted and examined with epifluorescence (Zeiss Axioskop) or confocal fluorescence microscope (Leica). The overlay pictures were created with the Adobe Photoshop software.

### Histological Characterization of Inflammation

**Figure 1:**
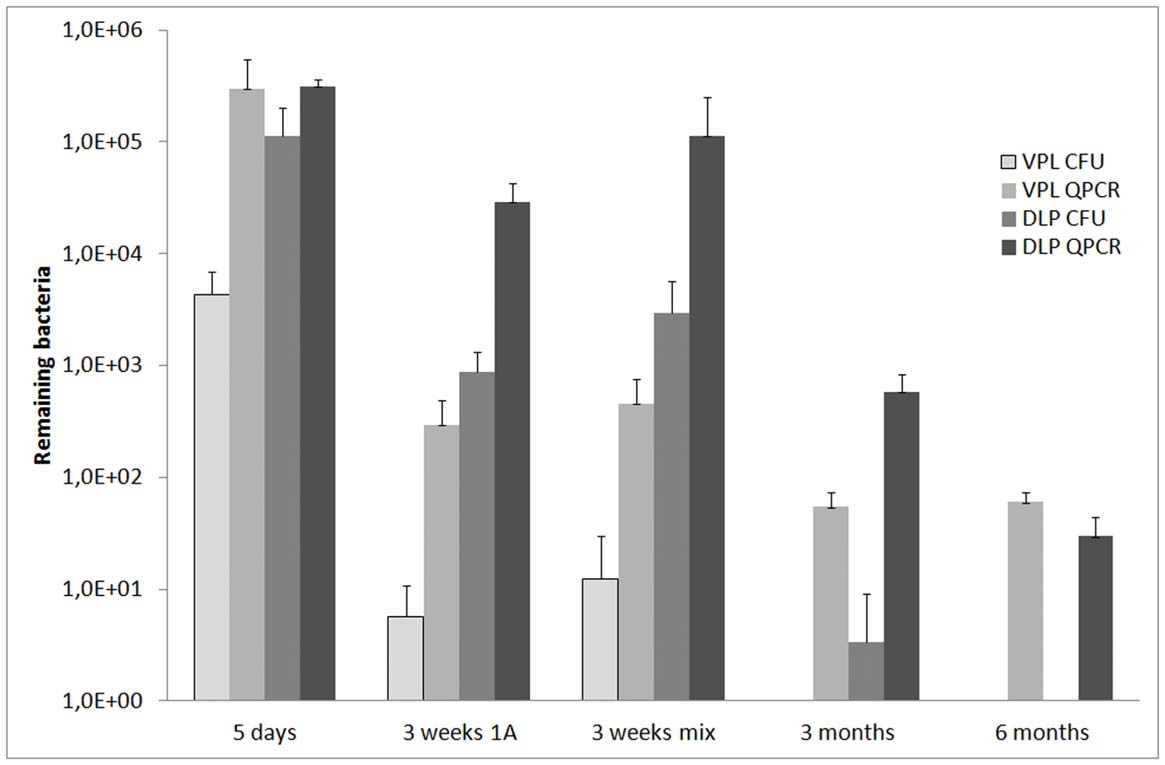
Inflammation scores. Panel of H&E-stained slides of infected prostate glands that illustrates the histological correlates of the inflammation score criteria, A) minimal inflammation, B) moderate inflammation, C) severe inflammation, D) focal inflammation, E) diffuse inflammation. Magnification: A–C: 100X, D–E: 40X.

Slides stained with hematoxylin & eosin were assessed microscopically. Inflammatory patterns were qualitatively categorized as focal or diffuse, and intensity of inflammation was scored based on the amount of infiltrating inflammatory cells. Patterns were categorized as diffuse when covering half the tissue or more, with no sharp borders between inflamed and non-inflamed regions. Patterns were categorized as focal when set off from surrounding normal tissue by clearly defined borders. Intensity of inflammation in each prostate specimen was categorized as minimal (≤5 leukocytes/5000 µm^2^), moderate (5–50 leukocytes/5000 µm^2^), or severe (> 50 leukocytes/5000 µm^2^), based on total leukocyte counts in five randomly selected 1000 µm^2^ areas. A panel of infected prostate glands illustrates the histological correlates of these criteria (Fig. 1). The volume of inflamed foci, as a percentage of total prostate volume, was determined microscopically by the method described in [18].

### Bacterial Counts and *P. acnes* Biotype Identification

Whole lobes were aseptically homogenized in PBS and 1/10 of the slurry obtained was subjected to 10-fold serial dilutions and plated on anaerobic blood agar plates. Plates were incubated for 1 week in 37°C under anaerobic atmosphere, whereafter *P. acnes* colonies were counted on the 1–2 plates with maximum resolution for each sample. Strains from biotype 1 & 2 differs in recA gene at base 71 (type 1 = G, type 2 = A), base 183 (type 1 = A, type 2 = G), base 214 (type 1 = C, type 2 = T) and base 424 (type 1 = A, type 2 = G). These SNPs were used to type the recovered isolates. Single colonies were transferred from the anaerobic blood agar plates into 100 µl PBS, and nucleic acid was prepared in a NorDiag Arrow preparation robot, using the Viral N/A extraction kit according to the manufacturer’s instructions (NorDiag AB, Hägersten, Sweden). 1 µl extracted DNA was template for a PCR reaction with primers parecAForw: AGCTCGGTGGGGTTCTCTCATC (0,3 µM) and parecARew: GCTTCCTCATACCACTGGTCATC (0,3 µM) [19], ABI SYBR Green PCR MasterMix (ABI, Warrington, UK), and water to a final volume of 25 µl. The PCR was performed on a 7900 HT Fast Real-Time PCR System, (Applied Biosystems) with a program consisting of: 95°C, 10 minutes (1 cycle), 95°C 60 s +50°C 30 s +72°C 90 s (35 cycles), 72°C 10 minutes (1 cycle). Sequencing of the PCR product was performed with parecAForw as primer (Eurofins MWG Operońs sequencing service, Eurofins MWG GmbH, Ebersberg, Germany). All sequences could unambiguously be assigned type 1 or type 2. QPCR assessment of *P. acnes* genome numbers were performed on the remains of the prostate lobe slurry, or, for the animals infected for 6 months that were not directly processed for CFU counts, whole lobes that were stored at −70°C until used. Tissue was disintegrated with a Fast Prep-24 sample preparation system with tubes containing Lysing Matrix M (MP Biomedicals, Solon,Ohio, USA), and nucleic acid was prepared in a NorDiag Arrow preparation robot, using the Viral N/A extraction kit according to the manufacturer’s instructions (NorDiag AB, Hägersten, Sweden). 5 µl (of total 50 µl) extracted DNA was template for a quantitative PCR reaction as described earlier [20]. A standard curve was created from VP lobes from non-infected rats spiked with a defined number of *P. acnes* bacterial cells (CCUG 41530) prepared from serial dilutions of a liquid broth culture (1.75 10^9^ CFU/ml). The standard curve, tissue processing and qPCR was performed as described above, and the Ct values vs CFU was plotted in a graph described by: CFU = 7E+13e^−0,914Ct^ (R^2^ = 0,9956). The calculations were performed with MS Excel.

### Preparation of Rabbit Anti-*Propionibacterium acnes* Polyclonal Antiserum

A washed suspension of *Propionibacterium acnes* in PBS corresponding to approximately 1 10^9^ cells/ml were treated with formaldehyde at a final concentration of 0.01 M. After incubation at 37°C on a slow shaker for 2 h followed by overnight shaking at room temperature, the bacteria were washed three times in PBS and then resuspended in PBS to an optical density of 1 and stored at 4°C. The inactivated bacterial culture was plated on anaerobic blood agar plates and incubated under anaerobic conditions for 10 days. No colonies were detected, indicating that the bacteria were completely inactivated. The inactivated bacteria were used as antigen to raise a polyclonal rabbit antiserum (Agrisera, Umeå, Sweden). The rabbits were maintained at the animal facility at Agrisera and all experiments involving animals were approved by the local Animal Review Board (Umeå, Sweden) (approval Id: A121-06).

### Blood Collection and Serological Methods

Blood samples were collected from rats by cardiac puncture prior to sacrifice. After coagulation and centrifugation at 1400 rpm for 10 minutes at room temperature, serum was collected. Presence of anti-*P. acnes* IgG was assessed by a western blot procedure where the rat serum functioned as the primary antibody. A total bacterial lysate (1·10^10^ bacterial cells dissolved in 400 µl Sample Buffer) was submitted to SDS-PAGE and electroblotted onto PVDF membrane. The filter was blocked by normal rat serum, cut into strips and incubated for 1 h with individual rat serum diluted 1∶2000. After washing, the strips were collected into a single container and incubated with goat anti-rat HRP antibody (1∶5000) for 1 h, washed and developed with ECC solution (Amersham). CRP levels were determined in an ELISA method (Rat Serum CRP M-1010) according to the manufacturer’s instructions (Alpha diagnostics, San Antonio, TX, USA).

### Statistical Analysis

Differences between interval variables and in average intensity scores of IgG were tested for by Student’s *t*-test. For small samples (*n* = 3) a permutation test was used [21]. Due to the small sample size, null hypotheses were rejected for p-values *equal* to 0.05 at the α = 0.05 significance level. Dependencies between categorical variables were tested for by Fisher’s exact test or its extension to larger tables.

## Results

### Prostatic Infection

**Figure 2:**
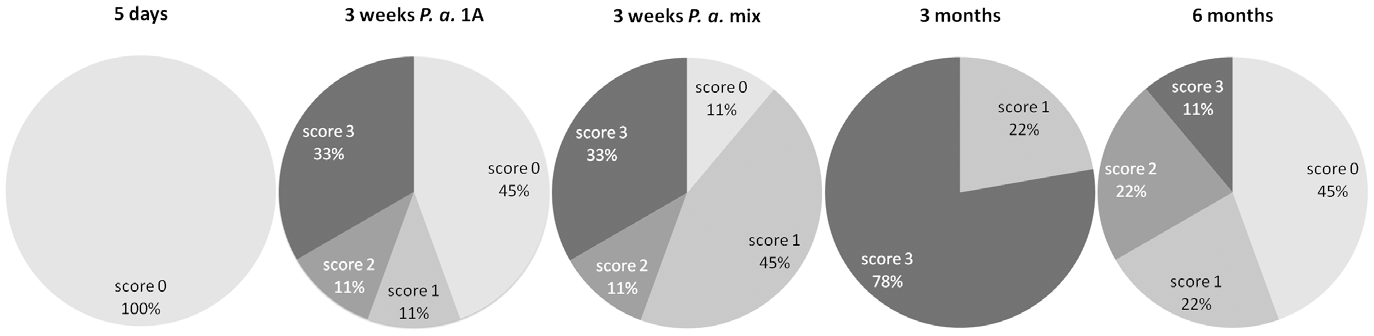
Recovered *P. acnes* during infection. Amount of *P. acnes* CFU and genome equivalents retrieved from left ventral prostate lobe (VP_L_) and dorso-lateral prostate lobe (DLP) 5 days, 3 weeks (1A), 3 weeks (mix), 3 months and 6 months after bacterial instillation. The number of CFU was determined by cultivation on solid agar and genome equivalents were determined by QPCR. Bars represent mean values, and the error bars represent standard deviation. (n = 2–3).

Tissue from VP_L_ and DLP in all experimental groups was assessed for the presence of viable *P. acnes* bacteria. Live bacteria were recovered from the prostate locales of infected animals throughout the duration of the experiment, with decreasing bacterial titers as infection times increased (Fig. 2). 5 days post infection, 6700, 1660 and 4600 CFU were recovered from VP_L_, and 60500, 215000 and 66000 from DLP. 3 weeks post infection, 10, 0 and 7 CFU were recovered from VP_L_ and 460, 790 and 1360 from DLP of the animals infected with *P. acnes* type 1A, and 32, 5 and 0 from VP_L_ and 860, 1900 and 6100 from DLP of the animals infected with the *P. acnes* mixture. 3 months post infection; no bacteria were recovered from VP_L_, and 0, 10 and 0 from DLP. Bacterial titer counts were significantly higher in DLP than in VP_L_ 5 days post infection (p = 0.05), 3 weeks post infection with type *P. acnes* 1A (p = 0.05), and 3 weeks post infection with *P. acnes* mixture (p = 0.05). The possibility that prostate-derived *P. acnes* isolates would be more potent than *P. acnes* of type 1A failed to find support in CFU recovery counts 3 weeks post infection (VPL: 1A < mix, p = 0.50, DLP: 1A < mix, p = 0.10). From the rats infected with the *P. acnes* mix for 3 weeks, 29 recovered bacterial clones, 12 of which from VP_L_ (2 individual rats), and 17 from DLP (3 individual rats), were analyzed for type. Of the VP_L_ isolates, 10/12 were of type 1 and 2/12 were of type 2. Of the DLP isolates, 5/17 were of type 1 and 12/17 were of type 2. Statistical testing for similarity in *P. acnes* type distributions between VP_L_ and DLP indicated difference (p = 0.00775; (1.53, 137.34) CI of odds ratio). 3 months post infection, 10 bacterial clones (all type 2) were recovered from the DLP of the only rat still harboring live bacteria. No *P. acnes* could be recovered from control animals. No bacterial count was performed 6 months post infection due to the low bacterial counts 3 months post infection. Quantitative PCR assessment showed presence of *P. acnes* genomes in both DLP and VP_L_ of all infected animals at 5 days, 3 weeks, 3 months and 6 months post infection. The number of genomes decreased with time after inoculation (Fig. 2). 5 days post infection, 270000, 70000 and 560000 genomes were detected in VP_L_, and 370000, 300000 and 270000 in DLP. 3 weeks post infection, 460, 80 and 350 genomes were detected in VP_L_ and 18000, 45000 and 24000 in DLP of the animals infected with *P. acnes* type 1A, and 750, 150 and 460 in VP_L_ and 270000, 11000 and 60000 from DLP of the animals infected with the *P. acnes* mixture. 3 months post infection, 60, 70 and 35 genomes were detected in VP_L_, and 300, 650 and 800 in DLP. 6 months post infection two rats were assessed: 50 and 70 genomes were detected in VP_L_, and 40 and 20 in DLP. Genome counts failed to differ between DLP and VP_L_ 5 days post infection (p = 0.45). However, 3 weeks post infection, counts were significantly higher in DLP than in VP_L_ in rats infected with *P. acnes* type 1A (p = 0.05), as well as in rats infected with *P. acnes* mix (p = 0.05). The possibility that prostate-derived *P. acnes* isolates would be more potent than *P. acnes* of type 1A failed to find support in genome counts 3 weeks post infection (VP_L_: p = 0.25, DLP: p = 0.2). Genome counts were significantly higher in DLP than in VP_L_ in rats infected for 3 months (p = 0.05). No hypothesis was tested regarding possible difference between DLP and VP_L_ 6 months post infection, due to the small number of rats.

### Serology

**Figure 3:**
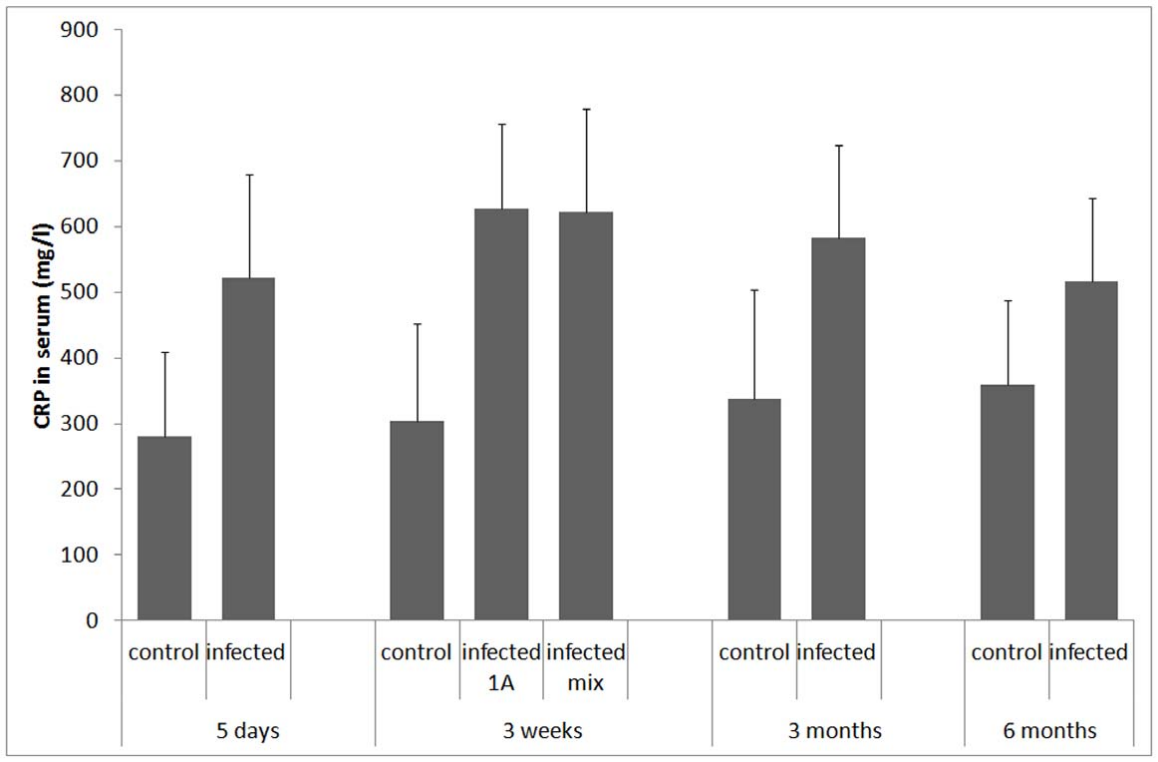
Rat serum content of anti-*P. acnes* IgG. Serum levels of anti-*P. acnes* IgG in rats 5 days, 3 weeks (1A), 3 weeks (mix), 3 months and 6 months after instillation. The level of anti-*P. acnes* IgG was scored in four steps, from 0 (non-detectable) to 3 (maximum level). The diagram shows the distribution of scores among infected rats (n = 9).

**Figure 4:**
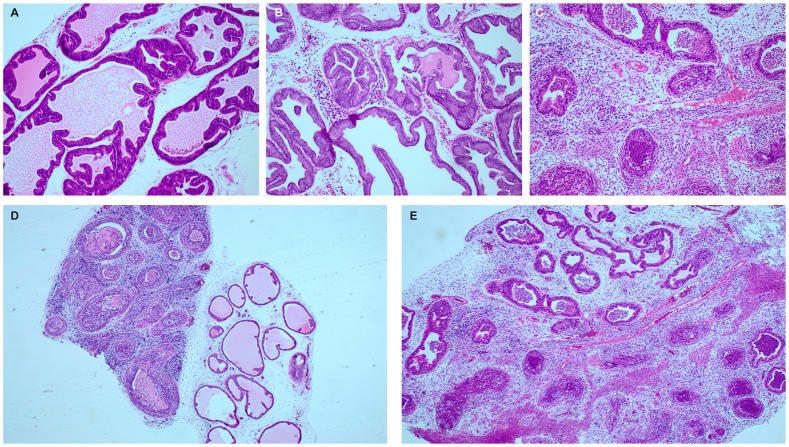
Serum levels of CRP. Serum levels of CRP in rats 5 days, 3 weeks 1A, 3 weeks mix, 3 months and 6 months after instillation with *P. acnes* and PBS control. Bars represent mean values, and error bars represent standard deviation (n = 9).

Aortic blood was collected when rats were sacrificed 5 days, 3 weeks (1A & mix), 3 months, and 6 months post infection. Blood serum from infected and control rats were assessed for presence of *P. acnes* binding IgG and rated 0–3, where 0 represents no *P. acnes* binding IgG (saline-instilled controls 5 days post infection), and 1–3 are positive in increasing degree. In the control group, all sera scored 0 at 5 days, 3 weeks, 3 months and 6 months post infection (data not shown). All sera scored 0 in the initial sample, 5 days post infection, but non-zero scores were found in subsequent samples (score distribution shown in figure 3). Tests indicated the highest average score in sera collected 3 months post infection (3 m>3 w: p = 0.04; 3 m>6 m: p = 0.0025). No score difference could be attributed to type of infective agent, 1A or mix, 3 weeks post infection (p = 0.45). Serum CRP was quantified 5 days, 3 weeks, 3 months and 6 months post infection. The infected animals had elevated CRP levels throughout the infection period, exhibiting an average of 1.86, 2.00, 2.13, 1.73 and 1.44 times the values of the control animals 5 days, 3 weeks (1A), 3 weeks (mix), 3 months and 6 months, respectively (Fig. 4). Statistical testing indicated difference between infected and controls in all groups (5d: p = 0.002, 3w 1A: p = 0.00, 3w mix: p = 0.00, 3 m: p = 0.02, 6 m: p = 0.02), but no difference could be attributed to type of infective agent, 1A or mix, 3 weeks post infection (p = 0.947).

### Histological Characterization of Inflammation

H&E-stained slides were scored for intensity of inflammation based on counts of infiltrating inflammatory cells, predominantly polymorphonuclear leukocytes in animals infected for 5 days, and lymphocytes in animals infected for 3 weeks, 3 months and 6 months, respectively, (two rats infected for 6 months showed an exception to this pattern and are described further below). Further, inflammation was categorized as focal or diffuse, and volume proportion of inflammatory foci to normal tissue was calculated using a stereological method.

### Ventral Prostate Lobe

**Figure 5:**
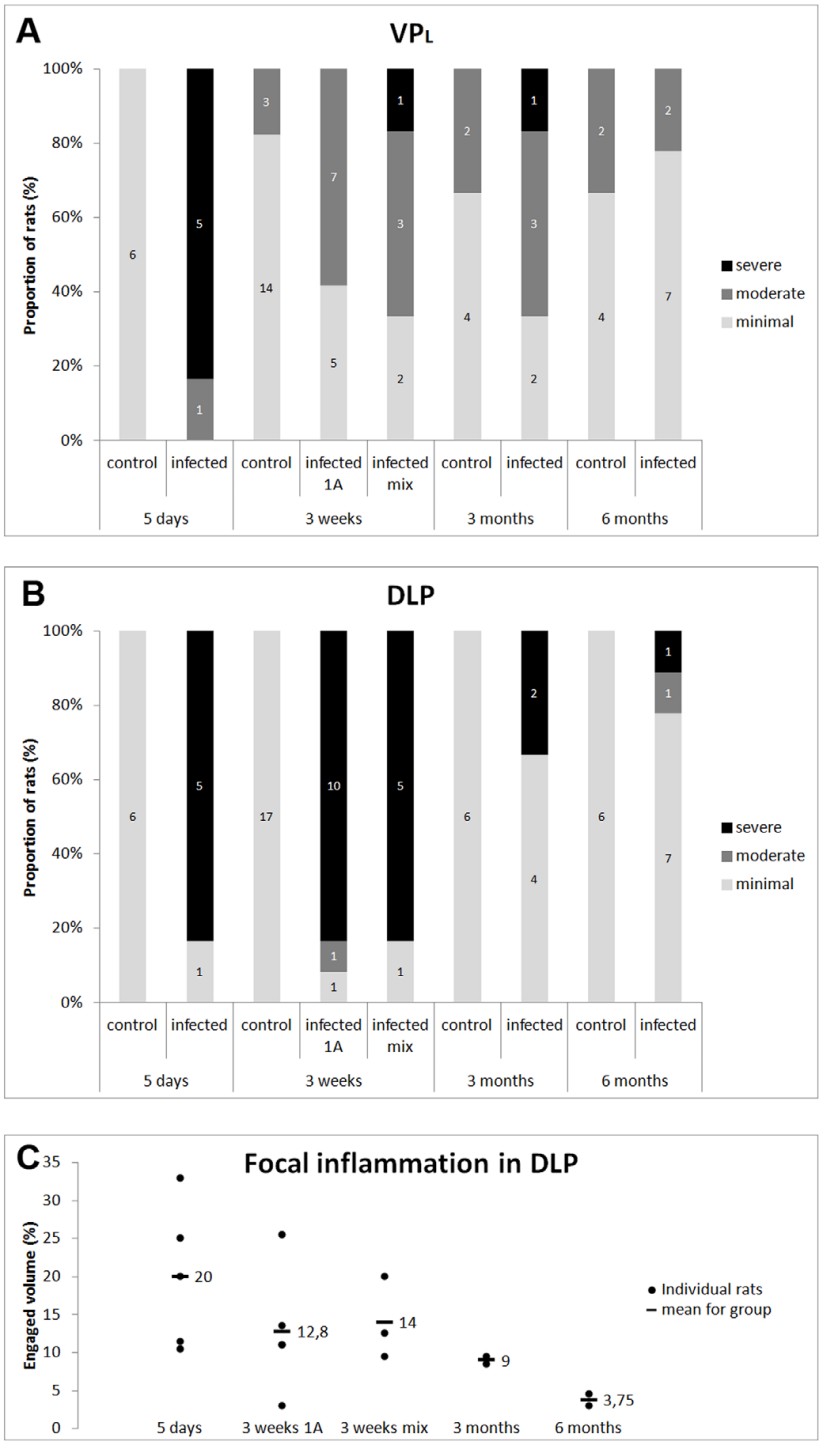
Degree of prostatic inflammation. Degree of inflammation in rats 5 days, 3 weeks (1A), 3 weeks (mix), 3 months and 6 months after instillation with *P. acnes* or PBS. Diagrams A & B show the distribution of scores among rats in each group, with n denoted in the bars. A) Left ventral prostate lobe (VPL), B) Dorso-lateral prostate lobe (DLP). C) Quantitative assessment of the spatial extent of focal inflammation in inflamed DLP. The diagram shows the individual distribution and means for the five groups (n = 5, 4, 3, 2, 2).

The left ventral prostate (VP_L_) exhibited severe diffuse inflammation 5 days post infection in 5/6 infected animals and moderate diffuse inflammation in 1/6 (Fig. 5A). No inflammation was seen in the lobe contra-lateral to the lobe injected with bacteria (VP_R_) (not shown) or in control animals (Fig. 5A). 3 weeks post infection, moderate diffuse inflammation was seen in VP_L_ in 7/12 of the animals infected with *P. acnes* type 1A, and in 3/6 animals infected with mix of prostate-derived *P.acnes* isolates. One of the animals infected with *P. acnes* mix had severe diffuse inflammation in VP_L_. Minimal diffuse inflammation was present in the VP_R_ in 4/18 of infected animals (not shown). In controls, 3/17 had minimal diffuse inflammation in VP_L_ (Fig. 5A), and 4/17 had mild diffuse inflammation in VP_R_ (not shown). 3 months post infection, moderate diffuse inflammation was present in VP_L_ in 3/6 (Fig. 5A) and in VP_R_ in 3/6 of infected animals. One rat (1/6) had severe diffuse inflammation in VP_L_. In controls, 2/6 animals had moderate diffuse inflammation in both VP_L_ (Fig. 5A) and VP_R_ (not shown). 6 months post infection, there was moderate diffuse inflammation in VP_L_ in 2/9 infected animals (Fig. 5A), and in VP_R_ in 1/9 (not shown). In controls, 2/6 had moderate diffuse inflammation in VP_L_ (Fig. 5A), and 1/6 had moderate diffuse inflammation in VP_R_ (not shown). Statistical analysis indicated significant difference between infected and control groups 5 days post infection (p = 0.002479), 3 weeks post infection (1A) (p = 0.04597), 3 weeks post infection (mix) (p = 0.045), but not 3 months (p = 0.5671) and 6 months post infection (p = 1). In addition, no significant difference could be established in inflammatory intensity between the two *P. acnes* agents 3 weeks post infection (p = 0.5362).

### Dorso-lateral Prostate Lobe

**Figure 6:**
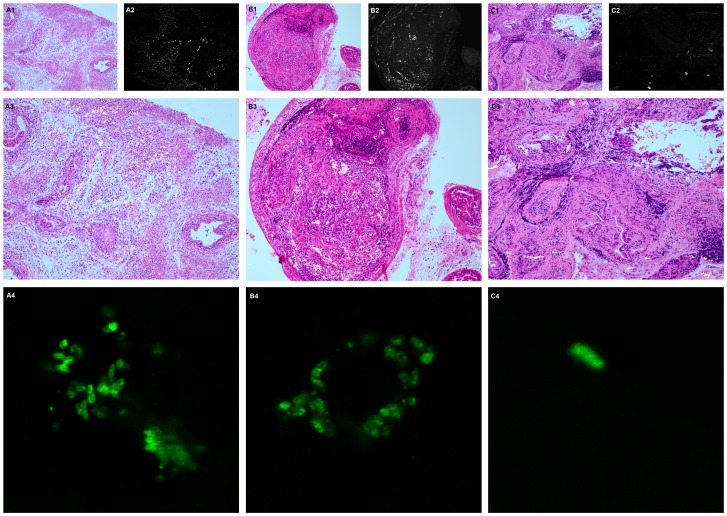
Corpora amylacea 6 months post infection. H&E-staining of inflamed foci in DLP 6 months after *P. acnes* instillation. Polymorphonuclear leukocytes are forming inflammatory infiltrate that surround structures resembling corpora amylacea. Magnification 400X.

In infected dorso-lateral prostate (DLP), inflammation was severe and focal in 5/6 animals, 5 days post infection. In these animals, 11–33% (mean: 20%) of the DLP volumes were inflamed (Fig. 5B+C). No inflammation was present in control animals. 3 weeks post infection, severe focal inflammation was present in 10/12, and moderate focal inflammation was present in 1/12 animals infected with *P. acnes* type 1A (Fig. 5B). Inflamed foci constituted 3–25% (mean: 12.8%) of the total DLP volume. Severe focal inflammation was present in 5/6 animals infected with *P. acnes* mix, and inflamed foci constituted 9–20% (mean 14%) of total DLP volume (Fig. 5 B+C). No inflammation was present in controls. 3 months post infection, severe focal inflammation was present in 2/6 infected animals. Inflamed foci constituted 8–10% (mean 9%) of the total DLP volume (Fig. 5 B+C). No inflammation was present in controls. 6 months post infection, severe focal inflammation was present in 1/9, and moderate focal inflammation in 1/9 infected animals (Fig. 5B). Inflamed foci constituted 3–5% (mean 3.75%) of the total DLP volume (Fig. 5 B+C). Uniquely for these animals, polymorphonuclear leukocytes formed the bulk of inflammatory infiltrate (Fig. 6). In one animal, structures resembling corpora amylacea were situated in conjunction with the inflammatory foci (Fig. 6).

Statistical analysis indicated significant difference between infected and control groups 5 days (p = 0.01515), 3 weeks (1A) (p = 0), 3 weeks (mix) (p = 0.0001783) post infection, but not 3 months (p = 0.4545) nor 6 months post infection (p = 1). There was no significant difference in inflammatory intensity between the two *P. acnes* agents 3 weeks post infection (p = 1).

### Visualization of *P. acnes* in Prostate Tissue

**Figure 7:**
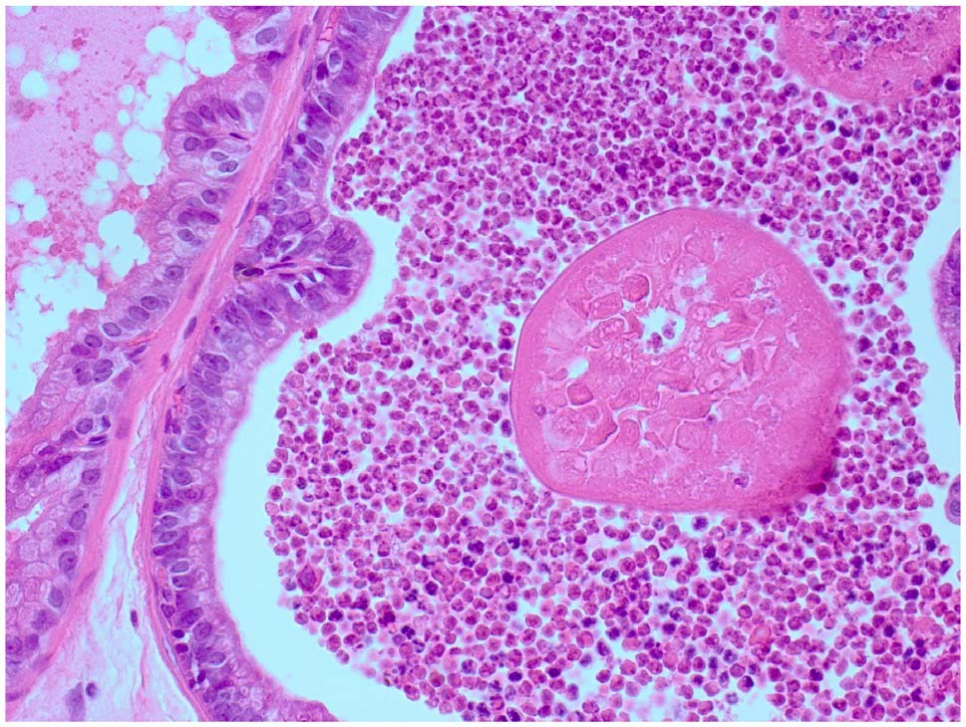
Visualization of *P. acnes* in infected prostate. Sequential sections of DLP from infected animals at 5 days (A), 3 weeks (B) and 3 months (C) after instillation, stained with H&E (1) and *P. acnes-* specific immunofluorescence (2). Pictures A3, B3 and C3 are created by overlaying 1 & 2. Pictures A4, B4 and C4 are close-ups of pictures A2, B2 and C2 captured with confocal microscopy. Magnification: 1–3∶100X, 4∶630X.

Sequential sections of VP_L_ and DLP from infected animals and controls were stained with H&E and *P. acnes-*specific immunofluorescence, respectively. Bacteria could be detected by microscopy in DLP up to 3 months and in VP_L_ up to 3 weeks post infection. Bacteria were seen both in stroma and in epithelial glands, and the presence of bacteria co-localized with foci of histological inflammation (Fig. 7). No bacteria were seen in controls.

## Discussion

We have established a novel rat model to investigate the pathogenic features of *P. acnes* infections of the prostate gland. Accumulating evidence for frequent presence of *P. acnes* in human diseased prostate tissue, combined with the widely accepted hypothesis that prostatic inflammation is a significant etiologic factor in both prostate cancer and benign prostatic hyperplasia (BPH), a characterization of the properties of this specific bacterial infection is highly relevant to the understanding of disease development. Instillation of 5·10^7^ CFU *P. acnes* into the prostate gland did not provoke any overt signs of disease, a fact that demonstrates the low direct pathogenicity of this bacterium. The infection initially caused a strong acute histological inflammation in both VP and DLP, and the inflammatory patterns were different in the two lobes; in DLP the pattern of inflammation was focal but in VP it was diffuse. In DLP the initial acute inflammation was succeeded by chronic inflammation that persisted in approximately 1/3 of the animals examined 3 months post infection. Bacterial cells were associated with inflammatory foci, and live bacteria could be retrieved from 1 out of 3 animals 3 months post infection. In VP, on the other hand, the initial acute state evolved into low grade inflammation. At an early stage, 3 weeks post infection, bacteria were only seen in a minority of animals, and only low numbers of live bacteria could be cultivated. The differences between lobes may suggest that the DLP is innately more susceptible for bacterial infections than is the VP. Similar conclusions have been drawn in studies of rodents experimentally infected with *E. coli* [22], [23]. Recent research suggests that clonal subpopulations of *P. acnes* carry specific virulence traits [24]. Studies have reported that *P. acnes* isolates derived from prostate are genetically and biochemically distinct from skin isolates, and that isolates derived from malignant prostate tissue are predominantly of type 2 [13], [17]. We infected rats with either prostate- or non-prostate derived *P. acnes* to observe possible differences in infectious or inflammatory properties between the bacteria. In addition, the prostate derived agent contained a mixture of 2 strains of biotype 1, and 2 strains of biotype 2. There were no differences between prostate and non-prostate isolates regarding severity of inflammation or remaining bacterial load 3 weeks post infection. However, the types distributed differently in prostate lobes; type 2 was more prone to persist in DLP. An interpretation of this result is that a wide range of *P. acnes* strains have capacity to exert the inflammatory effects observed at this time point, and, in addition, that properties specific to prostate-derived strains may have impact on locale tropism and infectivity. In animals infected with a mixture of isolates, only type 2 isolates could be recovered 3 months post infection. Although the number of recovered bacterial clones, 10 CFU, is too limited to support extensive conclusions regarding increased fitness for type 2 biotype in prostate infections, the result does not rule out this intriguing theory. The mean serum CRP levels of uninfected rats ranged between 280–360 µg/ml during the 6 months time span of the experiment. These values are in line with CRP levels reported for other rat strains [25]. The *P. acnes* infection caused an increase in serum CRP throughout the studied period, with a peak value of 627 µg/ml 3 weeks post infection. Elevated CRP levels, when prostatic inflammation has declined, are not typical for an acute-phase reaction [25]. The explanations may involve secondary infections, permanent tissue damage or intrinsic properties of this particular rat breed. The humoral immune response against *P. acnes* was maximal 3 months post infection, when all infected animals had detectable IgG, and a majority of them high titers. 6 months post infection, half the population had lost detectable *P. acnes* specific IgG, and only 10% had high titers. Sprague-Dawley rats are not prone to spontaneous development of prostatitis. The DLP is reported to stay free of histological inflammation up to one year of age [26]. Other studies report a 16% frequency of spontaneous prostatic inflammation in VP at 6 months of age [27]. Our results support these earlier reports in that the DLPs from our controls were free of inflammation throughout the study time up to 9–10 (3–4+6) months. VPs in controls exhibited spontaneous inflammation debuting at approximately 4 months of age, and at 9–10 months of age one third of animals were inflamed. Further studies are required to describe the molecular differences between prostate lobes responsible for this histological pattern. Recently, several mouse prostate infection models with uro-pathogenic *E. coli* have been published [23], [28], [29], [30]. Interestingly, the *E. coli* mouse models generate evidence of infection-dependant pre-cancerous tissue transformations [29], and reactive hyperplasia as well as increased epithelial proliferation [23]. Given the similarities in the prostatic inflammatory responses to *P. acnes* in our data and to *E. coli* infections reported in these studies, such adverse complications upon *P. acnes* infections of prostate may not be ruled out. Further studies are needed to assess the consequences of this chronic infection at cellular and tissue level. While it is beyond the scope of the present study to resolve the cellular identity of the inflammatory infiltrates, the observed aggregate of PMNs surrounding solid structures in the glandular lumen of DLP 6 months post infection (Fig. 6) may yet be of interest. Corpora amylacea (CA) inclusions in prostate glands are known to mainly consist of amyloid forms of proteins originating from neutrophil granules as reported by us and others [31], [32]. We suggest that figure 6 captures an early stage in the formation of CA, where neutrophiles have clustered, allowing their granule content to aggregate into a solid structure.

### Conclusions

*P. acnes* generate a self-limiting chronic inflammation in the prostate of rats. Thus, it is a potentially useful model system for general studies on long-term effects of infectious inflammation on prostate health. Upon *P. acnes* challenge, the prostate lobes differ in inflammatory response and bacterial clearance, a finding that is interesting to translate into a human system where predominantly the peripheral zone is affected by chronic inflammation.
