# Correction: Chronic Prostatic Infection and Inflammation by *Propionibacterium acnes* in a Rat Prostate Infection Model

**DOI:** 10.1371/annotation/2160e616-aa79-4097-96ab-e143d2a4d136

**Published:** 2013-07-31

**Authors:** Jan Olsson, Johanna Bergh Drott, Lovisa Laurantzon, Oscar Laurantzon, Anders Bergh, Fredrik Elgh

Multiple figures were accidentally switched. 

The image appearing as Figure 4 belongs with the title and legend for Figure 1, the image appearing as Figure 1 belongs with the title and legend for Figure 2, the image appearing as Figure 2 belongs with the title and legend for Figure 3, the image appearing as Figure 3 belongs with the title and legend for Figure 4, the image appearing as Figure 6 belongs with the title and legend for Figure 7 and the image appearing as Figure 7 belongs with the title and legend for Figure 6. 

